# Gender representation in science publication: evidence from *Brain Communications*

**DOI:** 10.1093/braincomms/fcac077

**Published:** 2022-03-25

**Authors:** Manuela Marescotti, Flavia Loreto, Tara L. Spires-Jones

**Affiliations:** 1 University of Edinburgh Centre for Discovery Brain Sciences, Edinburgh, UK; 2 Brain Communications Editorial Office, University of Edinburgh, Edinburgh, UK; 3 Department of Brain Sciences, Faculty of Medicine, Imperial College London, London, UK

**Keywords:** gender gap, neuroscience, STEMM, science publication, women in science

## Abstract

The persistent underrepresentation of women in Science, Technology, Engineering, Mathematics and Medicine (STEMM) points to the need to continue promoting the awareness and understanding of this phenomenon. Being one of the main outputs of scientific work, academic publications provide the opportunity to quantify the gender gap in science as well as to identify possible sources of bias and areas of improvement. *Brain Communications* is a ‘young’ journal founded in 2019, committed to transparent publication of rigorous work in neuroscience, neurology and psychiatry. For all manuscripts (*n* = 796) received by the journal between 2019 and 2021, we analysed the gender of all authors (*n* = 7721) and reviewers (*n* = 4492). Overall, women were 35.3% of all authors and 31.3% of invited reviewers. A considerably higher proportion of women was found in first authorship (42.4%) than in last authorship positions (24.9%). The representation of women authors and reviewers decreased further in the months following COVID-19 restrictions, suggesting a possible exacerbating role of the pandemic on existing disparities in science publication. The proportion of manuscripts accepted for publication was not significantly different according to the gender of the first, middle or last authors, meaning we found no evidence of gender bias within the review or editorial decision-making processes at *Brain Communications*.

## Teaser

The persistent underrepresentation of women in Science, Technology, Engineering, Mathematics and Medicine points to the need to continue promoting awareness and understanding of this phenomenon. At *Brain Communications*, we looked at the gender ratios of authors and reviewers of published manuscripts, aiming to examine whether our peer-review process is gender-biased.

## Introduction

The underrepresentation of women in Science, Technology, Engineering, Mathematics and Medicine (STEMM) disciplines has been reported worldwide.^1^ Increasing awareness of this issue has stimulated initiatives and discussion over the last decades, but equality is yet to be achieved and progress has proved slow so far.^1,2^

While the gender distribution tends to be approximately equal at graduate levels, the gap widens as career progresses.^3^ This phenomenon has been described as a ‘leaky pipeline’, to indicate that academia ‘leaks’ a higher number of women throughout career transitions towards more senior positions.^4^ Huang *et al.*^5^ found that women scientists have a 19.5% higher risk to leave academia than their male colleagues; for example, in the field of neuroscience women account for 31% of academic positions.^6^ A striking ‘case study’ of gender bias in neuroscience comes from the late, great glial expert Prof Ben (née Barbara) Barres who became a vocal advocate for women in STEMM after finding himself treated much better after transitioning from a female to male. If you have not read his commentary ‘Does gender matter,’ we highly recommend it.^7^

Being one of the main outputs of scientific work, research papers have been analysed by previous studies to estimate the gender gap in academic productivity and impact. In particular, men and women authors’ positions were analysed in line with prevailing conventions on authorship in science, where the order of authors reflects their seniority and contribution to the published work^8^ (see Box 1). Globally, women have been found to represent less than one-third of all authors in science,^9,10^ accounting for approximately 30% and 18% of first and last authors, respectively.^6,10^ Moreover, the underrepresentation of women seems to worsen in highly prestigious journals^3,10^ and citation rates are considerably higher for publications authored by male scientists.^5,9,10^ Although the gender gap appears to reduce every year,^3,10^ progress is still slow^1^ and preliminary reports suggest that the COVID-19 pandemic may have hindered this progress further by exacerbating existing disparities.^11^

**Box 1. Definition of roles of authors and reviewers according to prevailing conventions in science**

**Table:** 

First author	Author who generally performed most of the research in the paper and wrote the first draft
**Last (or senior) author**	Author who led the research financially and intellectually, often the principal investigator, a senior scientist or a lab lead.
**Corresponding author**	Author who can be contacted by the editorial team during submission/review or by the scientific community once the manuscript has been published. They can be of any seniority and position in the author list but are often the last authors.
**Middle author**	Author who contributed to the work generally either by doing parts of experiments or analyses, providing reagents or revising the paper but who did not have as much of a leading role as the first or last authors.
**Reviewers**	***Who are they?*** Scientists who actively involved in research are invited by associate editors to review manuscripts in their area of expertise. They can be at any career stage but are most often from postdoctoral and faculty levels. Their identity is usually hidden to the authors of the manuscript.
	***What do they do?*** They provide an objective evaluation of the manuscript (in the form of written comment) and advise the editorial office on whether to accept it, reject it or request minor or major revisions.
**Associate editors**	***Who are they?*** Research experts, often academics or postdoctoral researchers, expert in the disciplines published by the journal.
	***What do they do?*** They select reviewers, facilitate the peer-review process and make the final decision on publication based on their own and reviewers’ evaluation of the manuscript.

Data generated by submissions to peer-reviewed journals not only can inform on the gender gap in academic productivity and impact but also allows us to examine the possible contribution of editorial practices to the observed gap. Squazzoni *et al*.^12^ systematically examined three possible sources of bias (i.e. editorial selection of reviewers, reviewer recommendations, editorial decisions), each pertaining to three different stages of the peer-review and editorial processes, and found no evidence of gender bias in 145 scholarly journals.

*Brain Communications* is a ‘young’ open access peer-reviewed journal, founded in March 2019, committed to transparent, fair and author-friendly publication of rigorous work^13^ in the fields of neuroscience, neurology and psychiatry. Here, we analysed the gender of authors and reviewers of articles received by *Brain Communications*. The objectives were (i) to examine gender disparities in academic productivity and impact in neuroscience, neurology and psychiatry; (ii) to gain preliminary insight into the impact of the COVID-19 pandemic on the gender publication gap; and (iii) to assess whether peer-review and/or editorial decision-making at *Brain communications* directly or indirectly contribute to gender discrimination. These data are important for informing editorial decisions, such as the implementation of double-blind peer-review, to avoid gender bias.

## Methods

We included a total of 796 articles (774 non-commissioned and 22 commissioned) received by *Brain Communications* between mid-March 2019 and mid-October 2021, for which a final decision was made at the time of data collection. We analysed authors’ gender according to their position in the author list (first, middle and last), as well as the gender of all reviewers (*n* = 4492) invited to review non-commissioned content. In line with previous studies^3,10,14^ , we performed algorithmic estimation of gender from first names using the Genderize.io database.^15^

### Statistical analysis

Data were visualized and analysed in R Studio^16^. Chi-square tests were used to compare variables.

### Data availability

Data that support the findings of this study are available from the corresponding author, upon reasonable request.

## Results

### Is there a gender gap in academic productivity and impact?

We examined the gender of authors of all submissions, overall and according to their position in the author list. Moreover, we assessed whether women’s propensity towards involvement in peer-review differed from that of men.

#### Authors

**Figure 1:**
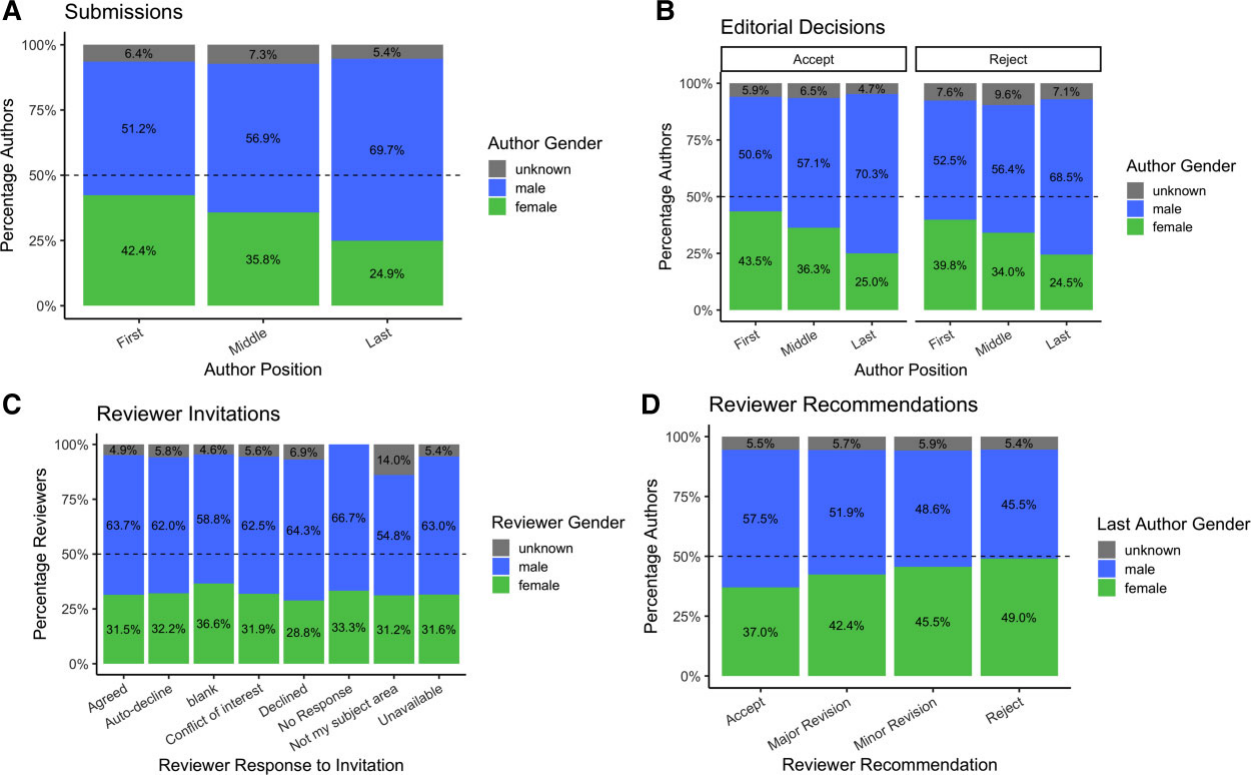
**Gender distribution of *Brain Communications* authors by position in the author list.** (**A**) Percentages of male, female and uncategorized authors in each authorship position in all submitted papers (*n* = 7722 authors). (**B**) Percentages of each gender in accepted (*n* = 5707 authors) and rejected (*n* = 2014 authors) papers did not reveal any evidence of bias in editorial decisions at *Brain Communications*. (**C**) Percentages of male and female reviewers invited to review papers are in line with the proportion of female neuroscience faculty members and last authors who submit to the Journal; we see similar response rates between men and women in agreeing or declining to review. (**D**) Percentage of reviewer recommendations (*n* = 1719 recommendations) examined by gender shows no significant difference in the proportion of positive versus negative recommendations for papers with a female last author.

Women constituted 35.3% of authors of all submissions (*n* = 796), and 42.4%, 35.8% and 24.9% of first, middle and last authors, respectively, with a significant difference in gender representation across the authorship categories (*c*^2^ = 66.31, d.f. = 4, *P* < 0.0001; Fig. 1A). This is in line with previous studies on high-profile neuroscience journals documenting more prominent gender publication gap for last (33.1%) than first authorship (18.1%).^10^

#### Reviewers

A comparable proportion of male and female reviewers invited by our Journal agreed to review manuscripts (30% and 30.5%, respectively; Fig. 1C), indicating similar attitudes of genders towards participation in peer-review.

### Gender and the COVID-19 pandemic

In March 2020, COVID-19 was declared a global pandemic^17^, and countries started enforcing lockdowns and social distancing measures to minimize the spread of the virus. A secondary effect of this was the suspension of non-essential research activities and the closure of laboratories, with detrimental consequences on research productivity. However, recent reports show that the magnitude of this effect may be bigger for women^14,18,19^, who are generally more involved in parenting and care duties.^14^ To gain insight into the possible exacerbating effects of COVID-19 on women’s underrepresentation, we examined whether the gender distribution of women authors and of available reviewers changed from the pre-pandemic period (March 2019 to March 2020) to the pandemic period (March 2020 to October 2021).

#### Author submissions

**Figure 2:**
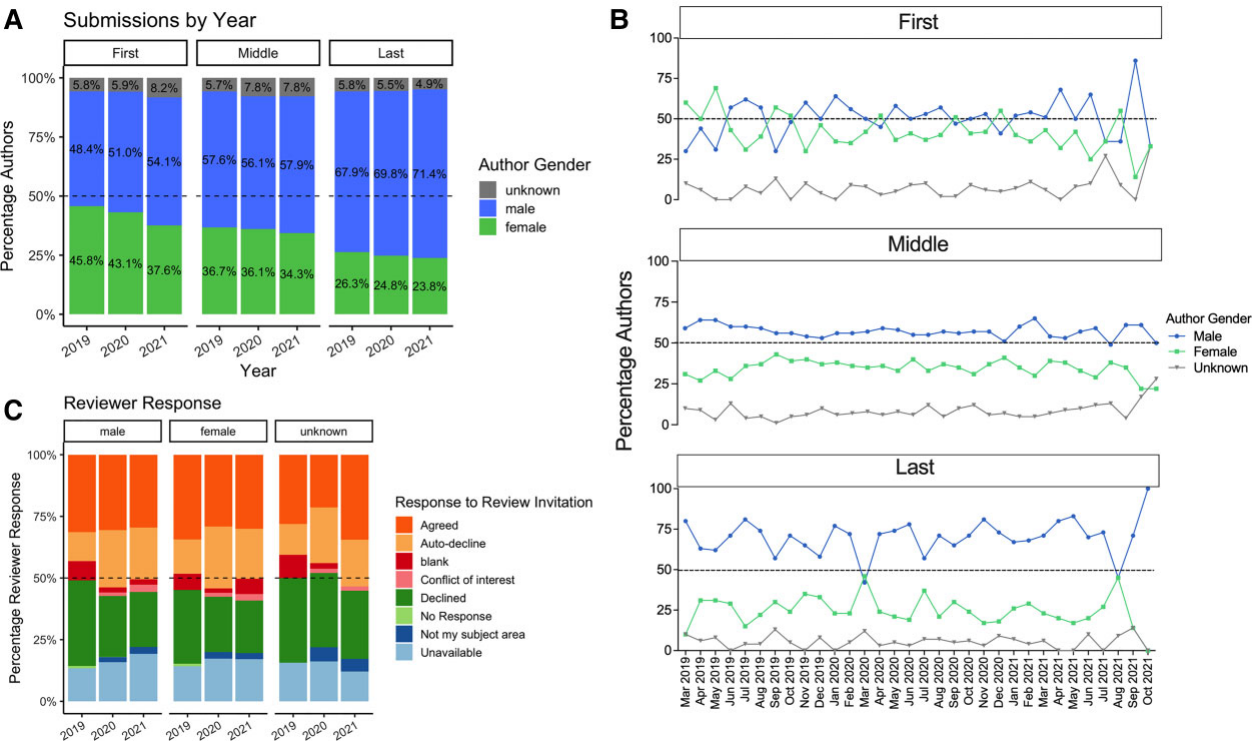
**Breakdown of gender distribution across 3 years, (A) yearly and (B) monthly for first, middle and last authors on all submissions (*n* = 796).** There was a downtick in female first and last authors at around the time many parts of the world went in to COVID-19 pandemic lockdowns. (**C**) Responses of female reviewers who were unavailable also increased and those who agreed to review decreased in 2020.

We observed a dip in the percentage of female authors in 2021 to 31.9% down from 36.2% in 2019, with the strongest impact seen among first authors which dropped from 45.8% in 2019 to 37.6% in 2021 (Fig. 2A). Trends varied across months and according to author type (Fig. 2B), and this may partly be due to different timings of COVID-19 waves and restrictions across countries.

#### Reviewer availability

The proportion of invited male reviewers who agreed to review papers decreased slightly from 2019 to 2020 (31.4% to 30.6%, respectively, leading to a drop of 0.8%). Women who agreed to review, instead, dropped more substantially by 5.3% (from 34.4% to 29.1%) in 2020 and recovered by 1% (to 30.1% agree responses) in 2021. Similarly, the proportion of both women and men who responded as ‘unavailable’ to review rose between 2019 and 2020 (by 3.1% for women and 2.5% for men) (Fig. 2C).

Although a definite cause–effect relationship cannot be established here, our data seem to corroborate a potential disproportionate effect of COVID-19 restrictions on women’s availability to both produce and review manuscripts. This may suggest that, during the pandemic, more women than men may have taken on different roles and responsibilities at the expenses of science productivity. Studies on gender gap in science publication will need to take this phenomenon into account going forward.

### Does *Brain Communications’* editorial decision-making contribute to the gender publication gap?

In this section, we examined whether *Brain Communications*’ editorial decision-making appeared to contribute to gender disparities at any of the three key steps of the peer-review process highlighted by Squazzoni *et al*.^12^

#### Editorial selection of reviewers

Firstly, to investigate whether the selection of reviewers was gender-biased, we compared the genders of 4492 reviewers invited to review 774 manuscripts. We observed that 31.2% of reviewers were women, 62.9% were men and 5.9% were classified as ‘unknown’ by the algorithm (Fig. 1C). This ratio was consistent across the years, with women accounting for 32.7%, 29.8% and 32.8% of invited reviewers in 2019, 2020, and 2021, respectively. This is in line with the proportion of submitting authors and with the proportion of women faculty members in neuroscience.^20^

#### Reviewer recommendations

Secondly, to examine whether the gender of authors seemed to influence manuscript assessment, we analysed the association between reviewers’ recommendations and last authors’ genders. We found that reviewer recommendations did not introduce a significant gender bias, although there were slightly lower percentages of female last authors on papers that received more positive (minor revision or accept) than more negative (reject or major revision) recommendations (Fig. 1D; *c*^2^ = 4.65, d.f. = 6, *P* = 0.59).

#### Editorial decision-making

Thirdly, to investigate a possible effect of authors’ gender on the likelihood of manuscript acceptance for publication, we separately tested the association between gender (male *versus* female) of the first, middle and last authors and editorial decisions (accept *versus* reject). Chi-squared tests revealed no overall difference in the proportion of accepted manuscripts according to the gender of first (*c*^2^ = 1.39, d.f. = 2, *P* = 0.50), middle (*c*^2^ = 0.66, d.f. = 2, *P* = 0.96) or last authors (*c*^2^ = 1.85, d.f. = 2, *P* = 0.40), indicating that editorial decisions did not appear to introduce gender bias (Fig. 1B).

## Discussion

This field potential analysed the representation of female authors in neuroscience, neurology and psychiatry through articles received by *Brain Communications*, a ‘young’ open access peer-review journal publishing robust and rigorous studies, committed to transparency in publication and promoting participation of a diverse community of scientists in the field.^13^

We found that 35.3% of all authors submitting to *Brain Communications* were women. This was higher than the 29.8% found by Bendels *et al*.^10^ in journals listed in the Nature Index or the 30% found by Larivière *et al*.^9^ in the whole area of science, but still suggestive of substantial gap. The analysis of authors’ positions revealed that women were considerably underrepresented as last-named authors (24.9%) but not as first-named authors (42.4%). This discrepancy has been consistently highlighted by previous studies^1,6,10^ and might indicate that women are more likely to be represented in junior than senior positions in academia. It is possible that this gap will decrease in the future, with more of today’s women early career researchers progressing to senior roles.^10^ Indeed, around 50% of neuroscience trainees are women, but only around 30% of neuroscience faculty members are women, which indicates that our submitting author pool is in line with the wider pool of neuroscience authors.^20^ However, progress is still slow and likely threatened by the COVID-19 pandemic.

Initial hints of a disproportionate impact of pandemic measures on women’s academic output were provided by the analysis of preprints^19,21^ and confirmed by a significant drop in women’s authorship in COVID-19 publications^11,14^ as well as in other research fields.^18,22^ Although our data are preliminary, submissions to *Brain Communications* in the years following the start of the pandemic did decrease for women but not for men in 2020 and in 2021. Notably, we also observed a larger drop in the availability of women reviewers in 2020. Future longer term studies are needed to investigate the possible reasons behind this phenomenon (e.g. higher involvement of women in caring duties or in other academic activities like teaching during COVID-19) and the role of confounders, such as geographical locations and severity of lockdown measures.

A third objective of the present work was to perform a critical evaluation of the editorial decision-making at *Brain Communications* to identify possible sources of bias. About one-third of invited reviewers were women, and they were as likely as men to agree to review a manuscript. Our percentage was higher than the ∼20% found by previous studies on other journals^23,12^, but slightly lower than the 35.3% of women authors who submitted to *Brain Communications*, highlighting a possible area of improvement for the Journal. However, it is worth noting that reviewers are more often senior scientists than first authors, due to their broader experience in the review process, and our invitation of female reviewers was higher than our proportion of female last authors. We did not find an association between authors’ genders and reviewers’ recommendations or between authors’ genders and editorial decisions, suggesting no systematic gender bias.

Overall, results of this work highlight persistent underrepresentation of women in publication of neuroscience research, especially when it comes to more senior roles. Our data do not allow us to draw conclusions on the underlying causes of this gap but further support the necessity to promote awareness and understanding of this phenomenon. Casad *et al.*^2^ recently discussed possible causes and solutions, illustrating the complexity of the problem and the need for multilevel interventions. In this context, we believe that journals can actively contribute to the progress towards gender equality in academia.

### Exercising positive action

Being the main channel for the dissemination of science, peer-review journals are well placed to study gender gap in academic publications and identifying its possible sources. Critical appraisal of the internal editorial processes can help targeting bad practices and finding areas of improvement. In light of the results of this study, *Brain Communications* will aim to improve the representation of women among reviewers through actions like our ‘Reviewer Academy’ detailed below. Inviting more women to review papers may promote gender equality within peer-reviewing but, more importantly, can improve gender diversity in a pool of specialized scientists which may also have more chances to be commissioned papers. In fact, reviewers can be invited by editors to write commentaries about manuscript they have reviewed. Holman *et al*.^1^ estimated that men are 1.7–2.1 times more likely than women to be invited to submit papers. At *Brain Communications*, 17 articles had been commissioned at the time of data collection, and these had 47% and 41% of women as first and last authors, respectively. One more action to address the underrepresentation of women in neuroscience comes in the form of training and career development opportunities. In 2021, *Brain Communications* launched two initiatives: the ‘Reviewer Academy’ and the ‘Observers Programme’. The Reviewer Academy aims to introduce and train early career researchers coming from a wide range of institutions and countries to the world of peer-review. One-hundred fifteen scientists have taken part in the academy to date, and 57% of them were women. The Observer Programme, instead, gives neuroscientists at any career stage the opportunity to shadow our scientific editor for half a day in order to gain greater understanding of how a peer-reviewed journal works and how to write effective papers. Moreover, this will also increase awareness of different career options in science publishing. With these and future initiatives, *Brain Communications* is committed to increase the participation of diverse, young and multicultural community to science.

### Limitations

Data showed in this paper contribute to the existing knowledge on gender representation in neuroscience. However, some limitations should be considered. The algorithmic estimation of gender from first names assumes a ‘gender binarism’ which belongs to the previous century.^24^ Moreover, in examining the peer-review process at *Brain Communications*, our work did not consider the possible role of authors’ affiliations which may be a source of bias in the editorial process.

## References

[1] Holman L , Stuart-Fox D, Hauser CE. The gender gap in science: How long until women are equally represented? PLoS Biol. 2018;16(4):e2004956. 29672508 10.1371/journal.pbio.2004956 PMC5908072

[2] Casad BJ , Franks JE, Garasky CE, et al Gender inequality in academia: Problems and solutions for women faculty in STEM. J Neurosci Res. 2021;99(1):13–23. 33103281 10.1002/jnr.24631

[3] Shen YA , Webster JM, Shoda Y, et al Persistent underrepresentation of women’s science in high-profile journals bioRxiv 2018;275362.

[4] Shaw AK , Stanton DE. Leaks in the pipeline: separating demographic inertia from ongoing gender differences in academia. Proc R Soc B Biol Sci. 2012;279(1743):3736–3741. 10.1098/rspb.2012.0822 PMC3415898 22719028

[5] Huang J , Gates AJ, Sinatra R, et al Historical comparison of gender inequality in scientific careers across countries and disciplines. Proc Natl Acad Sci U S A. 2020;117(9):4609–4616. 32071248 10.1073/pnas.1914221117 PMC7060730

[6] McDermott M , Gelb DJ, Wilson K, et al Sex differences in academic rank and publication rate at top-ranked US neurology programs. JAMA Neurol. 2018;75(8):956–961. 29610899 10.1001/jamaneurol.2018.0275 PMC6142929

[7] Barres BA . Does gender matter? Nature. 2006;442(7099):133–136. 16848004 10.1038/442133a

[8] Tscharntke T , Hochberg ME, Rand TA, et al Author sequence and credit for contributions in multiauthored publications. PLoS Biol. 2007;5(1):e18. 17227141 10.1371/journal.pbio.0050018 PMC1769438

[9] Larivière V , Ni C, Gingras Y, et al Bibliometrics: Global gender disparities in science. Nature. 2013;504:211–213. 24350369 10.1038/504211a

[10] Bendels MHK , Muller R, Brueggmann D, et al Gender disparities in high-quality research revealed by Nature Index journals. PLoS One. 2018;13(1):e0189136. 29293499 10.1371/journal.pone.0189136 PMC5749692

[11] Gabster BP , van Daalen K, Dhatt R, et al Challenges for the female academic during the COVID-19 pandemic. The Lancet. 2020;395(10242):1968. 10.1016/S0140-6736(20)31412-4 PMC7302767 32563275

[12] Squazzoni F , Bravo G, Farjam M, et al Peer review and gender bias: A study on 145 scholarly journals. Science Advances. 2021;7(2):eabd0299. 33523967 10.1126/sciadv.abd0299 PMC7787493

[13] Spires-Jones TL . Editorial. Brain Commun. 2019;1(1):fcz001. 32954256 10.1093/braincomms/fcz001 PMC7425211

[14] Pinho-Gomes AC , Peters S, Thompson K, et al Where are the women? Gender inequalities in COVID-19 research authorship. BMJ Glob Health. 2020;5(7):e002922. 10.1136/bmjgh-2020-002922 PMC7298677 32527733

[15] Genderize.io [Internet]. Roskilde, Denmark: Demografix ApS. [cited 2020 Dec 12]. Available from: <https://genderize.io/>.

[16] RStudio Team . RStudio: Integrated Development Environment for R. RStudio. PBC; 2020. http://www.rstudio.com/.

[17] Cucinotta D , Vanelli M. WHO Declares COVID-19 a Pandemic. Acta Biomed. 2020;91(1):157–160. 32191675 10.23750/abm.v91i1.9397 PMC7569573

[18] Ribarovska AK , Hutchinson MR, Pittman QJ, et al Gender inequality in publishing during the COVID-19 pandemic. Brain Behav Immun. 2021;91:1–3. 33212208 10.1016/j.bbi.2020.11.022 PMC7670232

[19] Inno L , Rotundi A, Piccialli A. COVID-19 lockdown effects on gender inequality. Nature Astronomy. 2020;4(12):1114–1114.

[20] Metitieri T , Mele S. Women in neuroscience: A short time travel. Ref Module Neurosci Biobehav. Psychol 2022:71–76.

[21] Viglione G . Are women publishing less during the pandemic? Here’s what the data say. Nature. 2020;581:365–366. 32433639 10.1038/d41586-020-01294-9

[22] Upthegrove R , de Cates A, Shuttleworth A, et al Gender equality in academic publishing: Action from the BJPsych. Br J Psychiatry. 2021;218(3):128–130. 33234181 10.1192/bjp.2020.192

[23] Steinberg JJ , Skae C, Sampson B. Gender gap, disparity, and inequality in peer review. The Lancet. 2018;391(10140):2602–2603. 10.1016/S0140-6736(18)31141-3 30070217

[24] Kullmann DM . Editorial. Brain. 2018;141(1):1. 29325050 10.1093/brain/awx345

